# A Sentence-Level Joint Relation Classification Model Based on Reinforcement Learning

**DOI:** 10.1155/2021/5557184

**Published:** 2021-05-26

**Authors:** Zhen Liu, XiaoQiang Di, Wei Song, WeiWu Ren

**Affiliations:** ^1^School of Computer Science and Technology, Changchun University of Science and Technology, Changchun, China; ^2^Jilin Key Laboratory of Network and Information Security, Changchun University of Science and Technology, Changchun, China; ^3^Information Center, Changchun University of Science and Technology, Changchun 130022, China

## Abstract

Relation classification is an important semantic processing task in the field of natural language processing (NLP). Data sources generally adopt remote monitoring strategies to automatically generate large-scale training data, which inevitably causes label noise problems. At the same time, another challenge is that important information can appear at any place in the sentence. This paper presents a sentence-level joint relation classification model. The model has two modules: a reinforcement learning (RL) agent and a joint network model. In particular, we combine bidirectional long short-term memory (Bi-LSTM) and attention mechanism as a joint model to process the text features of sentences and classify the relation between two entities. At the same time, we introduce an attention mechanism to discover hidden information in sentences. The joint training of the two modules solves the noise problem in relation extraction, sentence-level information extraction, and relation classification. Experimental results demonstrate that the model can effectively deal with data noise and achieve better relation classification performance at the sentence level.

## 1. Introduction

Relation classification is an important issue in natural language processing. It is mainly used in information extraction, knowledge graph construction [1], question answering system [2], and so on. Its task is to establish semantic relations between noun pairs. The definition of relationship classification is as follows. Given a sentence S, a pair of labelled entities *e*1 and *e*2, its purpose is to identify the relationship between e1 and e2. A piece of data in the dataset is cited to illustrate the specific function of relationship classification, for example: “to some experts on Europe, Slovenia is almost a model new member of the union.” In these data, the “contains” relationship between the noun Europe and Slovenia (place name) and the sentence itself is included. The purpose of relation classification is to identify the annotated entities in sentences and classify relations.

During the development of relation classification, experts have proposed various relation classification methods. Early methods are mainly based on named entity recognition (NER) [3] or use natural language processing systems to generate lexical features. Multi-instance and multilabel methods are used to deal with the problem of feature dependence and relationship overlap. These methods require experts to carefully select features and are limited by manpower and material resources. And in the process of extracting features, it is particularly easy to produce noisy data, which have a great impact on the computational cost and performance of downstream tasks. With the emergence of deep learning and distant supervision methods, the abovementioned problems have been better solved, but there are still many problems. The distant supervision method will bring a lot of noisy data when processing the data. Although the deep learning method can reduce the number of features extracted by experts, it still relies on vocabulary resources to obtain advanced features. It can be seen that the problem is mainly distributed in two aspects: noise data and feature extraction. From these two aspects, we will introduce different relationship classification methods.

In recent years, relationship classification problems mainly use deep neural networks to construct features, which can extract appropriate semantic features according to specific problems and no longer rely on experts to manually extract features. Moreover, in relation extraction tasks, the performance of neural network-based methods is better than that of earlier methods based on feature engineering. There are two main methods based on neural networks, one is recurrent neural network (RNN) [4] and the other is convolutional neural network (CNN) [5], both of which can train end-to-end neural network models. However, each has its advantages and disadvantages. Zeng At et al. [6] improved the CNN model, used a piecewise convolutional neural network (PCNN) to learn sentence representation, and only selected one effective sentence with the highest probability to train the relation extractor. However, the convolutional neural network is limited by the size of the convolution kernel and has certain limitations in the learning of sequence features. In addition, the convolutional neural network has low efficiency in classifying long sentence relationships. Although the Bi-RNN used by Zhou et al. [7] can access the previous and current information and increase the model's ability to process contextual information, the existence of the problem of gradient disappearance limits the scope of the model's access to the context. Both types of neural networks face the same problem. During the input process, the model will amplify sentence noise, thereby affecting the experimental results. In addition, models based on attention mechanisms such as sentence attention or word attention also perform well in relation classification. The sentence attention model proposed by Lin [8] and the word attention model proposed by Jat et al. [9] have different degrees of improvement on the original basis.

Generally, the training of neural network models requires a lot of labelled data. Although distant supervision can generate a lot of training data, the training data will be mixed with a lot of noise data. Reinforcement learning is a general method of learning the optimal solution through trial and error search and delayed rewards, which can effectively deal with noisy data. With the development of reinforcement learning in the field of NLP, reinforcement learning has been applied to relation extraction tasks by many experts and scholars. Zeng et al. [10] used a reinforcement learning model for large-scale relationship extraction and proposed a new reinforcement learning model, which successfully solves the problem of unclear sentence representation relationships in distant supervision datasets. Zeng et al. [11] applied reinforcement learning to the sentence relation extractor, following the hypothesis of at least one expression, and determined the delayed reward of reinforcement learning. Feng et al. [12] proposed a sentence-level relationship classification model based on noisy data, which uses RL to remove noisy sentences and selects high-quality sentences to train the relationship classifier. Qin [13] used RL to automatically identify noise data and divided noise data and useful data into different datasets. Joint relation extraction can also be modelled as a reinforcement learning problem. Feng et al. [14] modelled joint extraction as a two-step decision-making process, using the Q-learning algorithm as the model's strategy network to control the model's decision-making process. Takanobu et al. [15] modelled relation extraction as two levels of the reinforcement learning process and used a hierarchical reinforcement learning framework to extract relations and entities. Feng et al. [16] proposed a hierarchical RL model to solve the relation mention extraction problem. The model includes a high-level sentence selector to remove noisy sentences and a low-level relation mention extractor. S. Zheng et al. proposed a deep pattern diagnosis framework [17] for distant supervised relationship extraction. After the model merges the DS mark with the refined pattern, it has greatly improved in terms of identifying mark noise and relationship extraction performance. Reinforcement learning methods can also be applied to relational reasoning and intersentence relation extraction. Zhu et al. [18] used graph neural networks to process multihop relational reasoning. In terms of extracting the relationship between sentences, Sahu et al. used the GCN model on document-level graphs [19] to capture local and nonlocal dependent relation. Christopoulou et al. [20] proposed a neural network based on graph walking, which considers the interaction between multiple entity pairs in relation extraction. According to these methods, we are inspired to explore RL-based noise processing strategies.

Based on the feature extraction and noise processing methods mentioned above, we combine reinforcement learning and relationship classification and model the noise processing part of the relationship classification as a reinforcement learning process, and we use our proposed model to extract feature when processing data. We rely on the joint processing of the two parts to improve the accuracy of relationship classification and other indicators. Specifically, the model we proposed includes two parts: a joint network and an RL agent. The joint network is an end-to-end neural network based on the attention mechanism and the Bi-LSTM network. It is mainly used to extract useful semantic information in sentences, automatically select features that have an important impact on the relationship classification task, and predict the relationship of the sentence. In addition, we have added an attention mechanism to the joint network to improve the accuracy of relationship classification and the overall performance of the model. The main function of the RL agent is to identify and filter noisy sentences according to the policy network. In the training process, the RL agent and the joint network conduct joint training. In each interaction, the RL agent recognizes all sentences from the dataset, filters noisy sentences, selects high-quality sentences to input into the joint network, and updates its strategy network based on the rewards obtained from the joint network.

In summary, the contributions of this paper are as follows:

1. An end-to-end joint network model was designed to find hidden information in a sentence. The model includes Bi-LSTM and attention mechanisms. In joint training with the RL model, noisy data can be filtered without additional supervision.
2. The attention mechanism is introduced in the joint network module, and the attention layer is added after the Bi-LSTM layer so that the joint network obtains better performance.
3. Extensive experiments have been conducted on public datasets to evaluate the proposed method and discuss the results, and the experimental results demonstrated that the proposed method can achieve better performance than baselines.

## 2. Basic Theory and Concept

Reinforcement learning is a learning and decision-making calculation method; it is self-understanding, automatic, and goal-oriented. Reinforcement learning can map all situations encountered to its actions through learning to maximize the reward or numerical signal of the action. Different from other calculation methods, it emphasizes the interaction with the environment. It performs learning and decision-making in the interaction with the environment, without the need for exemplary supervision or complete environment modelling. It can be seen that reinforcement learning is learning in the process of interaction, and the two interacting parties are the agent and the environment, which are two essential elements in reinforcement learning. Reinforcement learning has two very important characteristics, namely, trial and error search and delayed reward. Trial and error search means that in the interaction between the agent and the environment, the agent is not told how to face the situation in the environment or what actions to take at the beginning, but in the interaction with the environment, it constantly tries to find those action which will get greater rewards. Delayed reward means that the immediate reward for an action taken in a certain situation cannot reflect the impact of this action on the entire decision-making process. Only after the decision-making process is over, can we know whether this action is useful or not in this situation. because the immediate reward will affect the subsequent decisions of the decision-making process.

First, we introduce the agent and environment. The modelling of the environment under different tasks is different. In this article, we treat datasets and federated networks as the external environment of reinforcement learning. Agent is a sentence selector, which selects sentences from the dataset and interacts with the federated network. The agent follows the set strategy to decide which action (select the current sentence or not) in each state (including the current sentence, the selected sentence set, and the entity pair), and then when all the choices are completed, delay rewards are obtained from the joint network model in the terminal state. Finally, in addition to the two elements of the agent and the environment, there are four subelements in the reinforcement learning system/framework: strategy, reward mechanism, value function, and model (not necessarily). We will introduce them separately in the following.

*Strategy*. In general reinforcement learning problems, the strategy may be random and nondeterministic. It usually gives the probability or probability distribution of the actions that can be performed. In this article, to ensure the decision-making and learning ability of reinforcement learning, the strategy is converted to the binary strategy *π*(*a*_*t*_*|s*_*t*_; *θ*) ∈ (0,1), which represents the probability distribution of a given parameter *θ* taking action *a*_*t*_ in the state *s*_*t*_:

(1) $$\begin{array}{c} \pi \left(a_{t}|s_{t};\theta \right)=F\left(Ws_{t}+b\right), \end{array}$$

where *θ*={*W*, *b*} are the parameters of the RL model strategy and F is the activation function.

*Reward Mechanism.* In the reinforcement learning problem, the environment and the agent must interact. In each step of the interaction, the environment returns a value to the agent, which represents the reward. Reward mechanism refers to a function defined in order to maximize the sum of total rewards during the entire interaction process. Therefore, the reward determines the quality of an action, but it does not mean that the bad action is bad throughout the interaction. The quality of the action is determined by the value function. Generally, the reward mechanism is represented by a random function of state S and action A.

In this article, the state S represents the current sentence. In order to obtain the potential information in the sentence, we first use word embedding and position embedding to convert the sentence into a vector form and then divide each feature map into three segments in the convolutional layer according to the position of the entity pair in the sentence. Finally, we use the max pooling operation on each fragment and concatenate the three outputs into a sentence representation. Meanwhile, when the RL model is training, the average value of each sentence vector representation will link up state as input to the model. Action means that the agent has two operations for each state: retention and filtering. For each sentence in the sentence set, the agent takes an action A to decide whether it should be retained. The value of the action is determined by the agent's strategy.

*Value Function*. The existence of the value function makes it clearer whether this action is good or bad throughout the interaction process. The value of a state is the sum of the immediate rewards accumulated from the beginning of the state to the end of the process. Although it may generate a very low immediate reward at a certain moment, it will have a high value after the interaction ends because it generates a large immediate reward at other moments. The reverse is also true. Analogous to us taking an exam, the level of immediate reward is equivalent to the test score of each subject. The score may be high or low, but the value given by the value function represents our total score in this test. In order to train the agent to obtain greater rewards during the interaction process, reinforcement learning introduces a value function. Therefore, what we care about is not the immediate reward that the agent gets at the current moment but the value function it gets in the decision-making process. The choice of actions is also based on the judgment and evaluation of the value function. In this article, we no longer use the value of the reward as the judgment of the action in the value function but use the Polity Gradient method instead. It does not calculate the value of the reward but uses the probability to select the action, which avoids the calculation of the reward and maintain the state table. The basic principle of policy gradient is to adjust the policy through environmental feedback, which refers to the probability of taking an action in the Polity Gradient method. Specifically, when a positive reward is obtained, the probability of the corresponding action is increased; when a negative reward is obtained, the probability of the corresponding action is reduced. Policy Gradient is a strategy that directly learns parameterization, and action selection no longer depends on the value function. The reward is maximized by applying a gradient ascent algorithm to the strategy function. Correspondingly in the dataset, taking an action for each sentence in sentence set B determines whether the sentence is selected. After all sentences are selected, the model obtains the final probability. Therefore, we only get delayed rewards in the final state. The definition of probability is as follows:

(2) $$\begin{array}{c} \pi \left(s_{i}|B\right)=\left\{\begin{array}{c} 0,\;i<\left|B\right|+1,\\ \frac{1}{\left|B\right|}\sum_{x\in B}\log\;p\left(r|x\right),\;i=\left|B\right|+1, \end{array}\right. \end{array}$$

where B is a set of selected sentences and *r* represents the relation label.

For each time position *t* in the sequence, the gradient ascent method is used to update the parameter *θ* of the strategy function:

(3) $$\begin{array}{c} \theta =\theta +\alpha \nabla_{\theta }\log\;\pi_{\theta }\left(s_{t},a_{t}\right)\nu_{t}. \end{array}$$

LSTM is a variant of recurrent neural network (RNN). It uses LSTM units to replace the hidden layer in RNN, which can effectively avoid the problem of the disappearance of the gradient of the neural network. LSTM is mainly composed of the memory part of the LSTM unit and three “gates.” The three gates are input gate (*i*), forget gate (*f*), and output gate (*o*). It is essentially a memory block in a neural network. The main idea of the LSTM unit is to introduce an adaptive gating mechanism, which determines the extent to which it retains the results of the previous moment at the current moment and remembers how many extracted features from the current input data.

**Figure 1:**
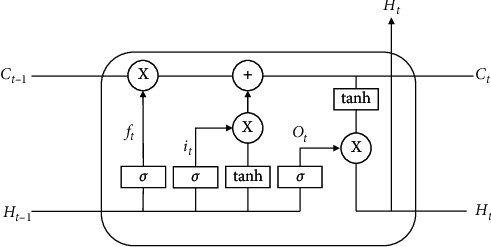
Structure of LSTM cell.

The Bi-LSTM network model used in this article is a combination of forward LSTM and backward LSTM. The calculation process of Bi-LSTM is essentially the same as the unidirectional long short-term memory (LSTM) network model. We take the forward LSTM as an example. As we can see in Figure 1, at each time step *t*, a LSTM cell calculates three parts of hidden vector information: current word embedding *w*_*t*_, previously hidden vector *h*_*t*−1_, and the previous cell vector *C*_*t*−1_. The specific details of the operation can be formalized as follows:

(4) $$\begin{array}{c} i_{t}=\;\delta \left(W_{i}\cdot \left[w_{t},h_{t-1},C_{t-1}\right]+b_{i}\right), \end{array}$$

(5) $$\begin{array}{c} f_{t}=\;\delta \left(W_{f}\cdot \left[w_{t},h_{t-1},C_{t-1}\right]+b_{f}\right), \end{array}$$

(6) $$\begin{array}{c} z_{t}=\;\tanh\left(W_{c}\cdot \left[w_{t},h_{t-1},C_{t-1}\right]+b_{c}\right), \end{array}$$

(7) $$\begin{array}{c} c_{t}=f_{t}*c_{t-1}+i_{t}*z_{t}, \end{array}$$

(8) $$\begin{array}{c} o_{t}=\;\delta \left(W_{o}\cdot \left[w_{t},h_{t-1},C_{t-1}\right]+b_{o}\right), \end{array}$$

(9) $$\begin{array}{c} \overset{⟶}{h_{t}}=o_{t}*\;\tanh\left(c_{t}\right), \end{array}$$

where W is the parameter, *b* is the bias, *c* is the cell memory, and · is vector multiplication.

Unlike LSTM, Bi-LSTM first adds the weight parameter matrix and bias vector corresponding to the hidden layer processing of the positive sequence for the hidden layer of the inverse sequence. The positive sequence and the inverse sequence will pass their respective weight parameter matrix and bias. The vector obtains the output vector of the hidden layer. Finally, the forward output vector and the backward output vector are merged. For different applications, their merge methods will be slightly different. In this article, we will use the connection method to combine the two output vectors and get the following results:

(10) $$\begin{array}{c} h_{i}=\left[\overset{⟶}{h_{i}};\overset{⟵}{h_{i}}\right]. \end{array}$$

## 3. Methods

**Figure 2:**
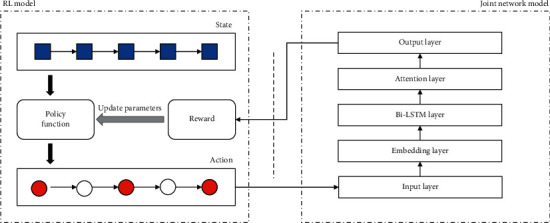
Joint network model and RL model.

The overall structure of the framework proposed in this paper is shown in Figure 2. The framework consists of two parts: a joint network model and a RL model. The joint network is an end-to-end model based on the attention mechanism and Bi-LSTM network. It is independent of the RL model. The function of the RL model is to remove noisy sentences from the original dataset and construct a clean dataset. Each sentence from the original dataset is determined by the agent's policy network, and the agent takes actions to decide whether the sentence should be deleted. After that, the agent filters the noisy sentences in the training dataset and then uses the dataset to train and update the joint network, and finally it calculates the reward in the final state and passes the reward of the RL network. At last, the RL agent can use the received rewards to update its policy network and initiate a new selection process.

In this paper, we take data denoting as a RL problem. The purpose is to obtain an agent that can take correct actions to remove noisy sentences according to the state of the external environment, and the obtained agent interacts with the external environment to get the rewards from the joint network and update their parameters. The joint learning of the two modules can reduce the noise data in the distant supervision process and effectively improve the classification performance.

### 3.1. The Joint Network

**Figure 3:**
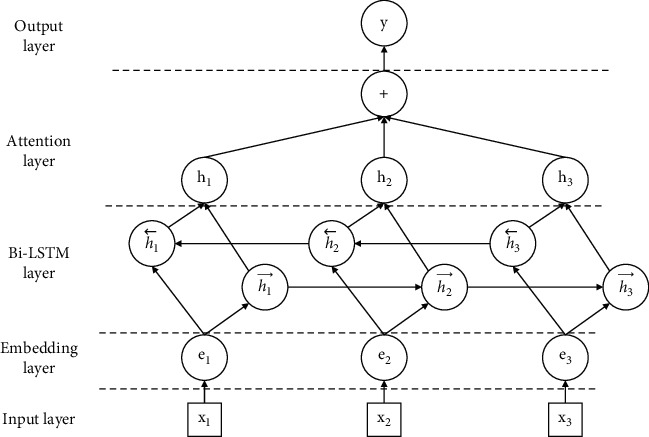
Structure of the joint network model.

The joint network model proposed in this paper is shown in Figure 3 and consists of five parts as follows:

(1) *Input Layer*. Sentences are given as input into the model.
(2) *Embedding Layer*. The role of the embedding layer is to represent sentences in vectors. Specifically, for each sentence *S* = {*x*_1_, *x*_2_, ..., *x*_*t*_} consisting of *T* words, we represent it as a vector list *E* = (*e*_1_, *e*_2_, ..., *e*_*t*_); each word *x*_*i*_ is converted into a representation vector *e*_*i*_. The vector includes two parts: position vector and word vector. Word embedding is obtained by looking up the word2vec embedding matrix. The purpose is to convert each word into a vector form with a dimension of *d*_*w*_. Position embedding includes a vector representation of the relative distance between a word and two entities in a sentence, and the dimension is *d*_*p*_, so it is embedded by two positions. Finally, we connect the word embedding of each word and the two position embeddings to form a new vector *e* (e∈*R*_*d*_, *d* =  *d*_*w*_ + 2 × *d*_*p*_) and then input these vectors into the Bi-LSTM layer.
(3) *Bi-LSTM Layer*. The sentence vector representation obtained by the embedding layer is given as input into the neural network, and Bi-LSTM is used to process the vector and obtain high-level features. It can mine hidden information in sentences through forward and backward LSTM and merge the two results into one result.
(4) *Attention Layer*. Attention mechanism is added to the result of Bi-LSTM layer. This layer can further dig out the hidden information in the sentence and add higher weight to the important information in the sentence. At the same time, in order to effectively distinguish the effective features and invalid features in the sentence, we have made some adjustments to the attention mechanism to build a new attention layer.
(5) *Output Layer*. Sentence-level feature vectors are used for relation classification, and the prediction probability of relation is calculated. In this layer, for each sentence S, we use a SoftMax classifier to calculate the predicted probability *p* of the relation *y*, and the separator takes *h*^*∗*^ from the attention layer as input. The relation is predicted as follows:
  (11) $$\begin{array}{c} p\left(y|S\right)=\text{softmax}\left(W^{\left(S\right)}h^{*}+b^{\left(S\right)}\right). \end{array}$$

In this article, we use cross entropy to define the objective function and use L2 regularization and dropout to alleviate overfitting. Dropout is proposed by Hinton et al. In the forward propagation process, the cooperative adaptation of hidden cell is prevented by randomly omitting feature detectors from the network. We use the dropout algorithm for the sentence representation obtained in the embedding layer, Bi-LSTM layer, and attention layer. The loss function is as follows:

(12) $$\begin{array}{c} J\left(\theta \right)=-\frac{1}{m}\sum_{i=1}^{m}\log\left(p\left(y_{i}|S_{i}\right)\right)+\lambda \theta_{F}^{2}, \end{array}$$

where *p* is a prediction probability of each relation, *m* is the number of target relation, and *λ* is the parameter of L2 regularization.

### 3.2. Attention

When a neural network processes a variable length vector sequence, we can usually use a convolutional network or a recurrent network to encode, and then we get an output vector sequence of the same length, whether it is using a convolutional neural network or a recurrent neural network or , in fact, both. It is a kind of “local encoding” for variable length sequences. There are two ways to solve this short-distance dependent “local coding” problem. One method is to use a fully connected network, and the other method is to increase the number of layers of the network and obtain long-distance information interaction through a deep network. We adopt a fully connected network to deal with the above problems. Although the fully connected network is a very direct model for modelling long-distance dependence, it cannot handle variable length input sequences. Because of different input lengths, the size of the connection weight is different. In this article, we adopt the self-attention mechanism. Since the weight of the self-attention mechanism model is dynamically generated, it can handle variable length information sequences. Putting the attention layer on the Bi-LSTM layer can increase the neural network's ability to process variable length information sequences.

In text classification tasks, the attention mechanism can be used to capture the connection between each unit (such as each word) in the input sequence and other units. Its essence can be described as a mapping from query *Q* to a series of key-value pairs (K, V). Generally speaking, for each LSTM decoder unit, we take the input *Z* of the previous decoder unit as the query vector *Q* and the hidden state output by the encoder as the key-value pair vector (K, V). Specifically, the vector *H* is first tangent and passed to the fully connected layer. Then, the model uses the *softmax* function to normalize the output of the fully connected layer and the context vector parameter *w* (random initialization) that can be trained, and finally the calibration coefficient *α* is obtained. The model multiplies the word-level features of each time step by the weight vector and merges them into sentence-level feature vectors. The attention vector *r* can be expressed as a weighted sum of all vectors. The context vector *w* can be interpreted as the best word represented on average. When the model faces a new sample, it uses this knowledge to determine which word needs more attention. During training, the model will update the context vector through backpropagation; that is, it will adjust the internal representation to determine what the optimal word is. Let *H* be a matrix [*h*_1_, *h*_2_, ..., *h*_*t*_] generated by output vectors composed by the Bi-LSTM layer, where *T* is the sentence length. The sentence representation *r* is weighted by these output vectors:

(13) $$\begin{array}{c} M=\tanh\left(H\right), \end{array}$$

(14) $$\begin{array}{c} \alpha =\text{softmax}\left(w^{T}M\right), \end{array}$$

(15) $$\begin{array}{c} r=H\alpha^{T}, \end{array}$$

where *w* is a trained parameter vector and *w*^*T*^ is its transposed matrix.

Finally, we get the sentence representation for classification as follows:

(16) $$\begin{array}{c} h^{*}=\tanh\left(r\right). \end{array}$$

## 4. Our Algorithm

In the method described in this paper, pretraining is very important. First, we pretrain the joint network model and the RL model, respectively, and after that we obtain pretrained parameters of the two network models and freeze them. Finally, we jointly train the RL model and the joint network model, as shown in Algorithm 1.

**Figure 4:**
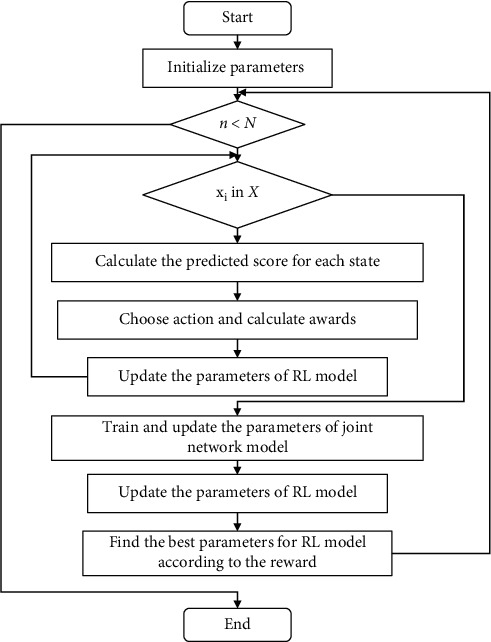
Flow chart of joint training.

As described in lines 2–4, for each training data *x*_*i*_, in order to calculate the action taken by the RL model on the state, the RL model calculates the prediction score of the state to obtain the action. Line 5 calculates temporary and average rewards, which are used to calculate delayed rewards and obtain optimal parameters based on rewards, such as lines 7 and 10. The parameters of the RL model are updated in line 6. After the calculation of the training data is completed, the parameters of the joint network model are trained and updated. At the same time, a new input is generated for the next round. Finally, the parameters of the RL model are updated, and the parameters of the new RL model are the optimal parameters. The specific process is shown in Figure 4. Especially during the joint training process, we add weight factor *λ* in parameter update to speed up the network update.

## 5. Experiment

### 5.1. Dataset and Evaluation Indicators

To evaluate our model, we conducted experiments on the New York Times (NYT) corpus. Since the corpus was provided by distant supervision, all the datasets contain noisy data. It composes of 52 positive relation classes and 1 negative relation class. The statistical information about the dataset is shown in Table 1.

In order to evaluate the effectiveness of the model, widely used indicators precision (P), recall (*R*), and F1-scores (F1) are used to measure the performance of the model. When the relation type and both entities are correct, the extracted triple is considered correct.

### 5.2. Experimental Setup

Our experiment was performed on a single Nvidia Titan X GPU using Python 3.6 and TensorFlow 1.6.0. As we can see in Table 2 the word embedding and position embedding, we used 50-dimension and 5-dimension. The batch size is 128, and the number of extractions each time is 3 times. We use the dropout algorithm for the sentence representations obtained in the embedding layer, Bi-LSTM layer, and attention layer, and the values of dropout are 0.5, 0.75, and 0.5. In the Bi-LSTM layer, the number of hidden cells set for Bi-LSTM is 128. We define the learning rate to 0.02, the update rate to 0.01, and the L2 regularization parameter to 0.0001. In the sentence representation, the sentence length is 70, the number of convolution kernels is set to 60, the step size is set to 1, and the output length is 230.

### 5.3. Experimental Results and Discussion

The experiment in this article is divided into three parts, including joint network model training, RL model training, and joint training. The training of the first two models is pretraining in order to converge as soon as possible for the joint model. In this paper, the comparison results of different methods are obtained through experiments, and the precision, recall, and F1-scores of each group of experiments are recorded, as shown in Table 3.

It can be seen that compared with LSTM, Bi-LSTM has a slight improvement in precision, recall, and F1-scores, which proves that Bi-LSTM is more effective than LSTM, although the training time of Bi-LSTM experiment is longer. The experimental result of Bi-LSTM-CRF and Bi-LSTM-Attn shows that the joint experimental results of the attention mechanism and conditional random field with Bi-LSTM are significantly improved in all aspects compared with the simple Bi-LSTM. Moreover, the performance effect of the Bi-LSTM model under the attention mechanism is better than that of the model with a conditional random field, which proves that the attention mechanism can further improve the performance of relation classification and at the same time proves the effectiveness of the joint network training model proposed in this paper. In joint training with the RL model, we conducted two sets of experiments. As shown in Table 3, compared with the traditional end-to-end model, the accuracy has been significantly improved. Therefore, the method based on RL can better deal with noisy data. Our proposed RL-based joint network model outperforms most methods.

**Figure 5:**
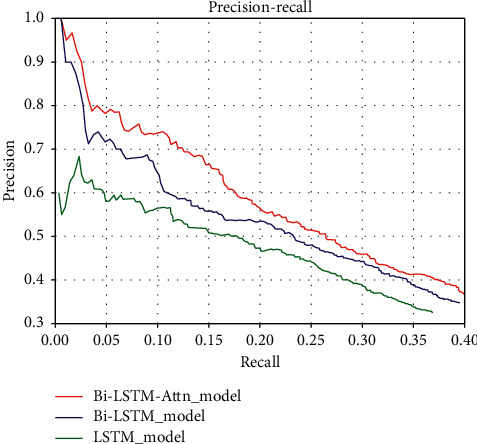
Performance comparison of different models.

We compared the performance of different models with the precision-recall curve as shown in Figure 5. Obviously, we can find that the traditional LSTM method performs much worse than the Bi-LSTM method, and the Bi-LSTM model with the added attention mechanism is much better than the original Bi-LSTM model. This shows the effectiveness of using attention mechanism to mine the hidden information of sentences in relation classification tasks with noisy labels.

**Figure 6:**
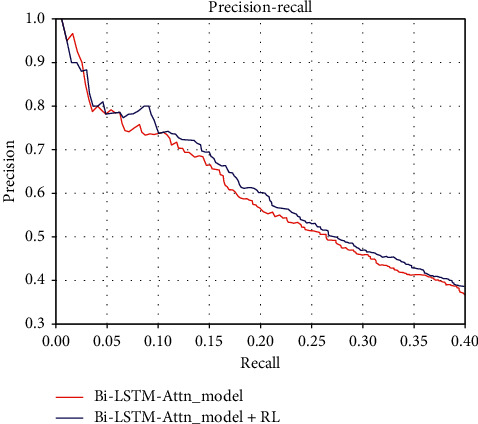
Performance comparison of joint network model with reinforcement learning against joint network model without reinforcement learning.

In order to verify the effectiveness of joint training with RL, we conduct experiments with only joint network models and experiments with joint network models with RL models. From Figure 6, we can observe that combining the RL mechanism can improve the performance of the original Bi-LSTM-Attn model. For this model, when the recall rate is greater than 0.05, the performance of the model using the RL mechanism is better than that of the original model. Therefore, based on RL method for removing noise data is valid, and the joint learning of the two modules can reduce the noise of the dataset and effectively improve the classification performance.

**Figure 7:**
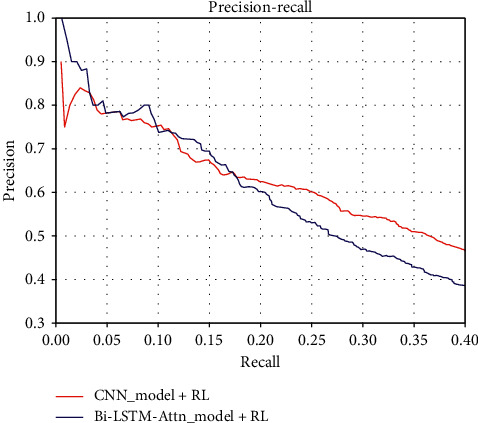
Performance comparison of joint network and CNN network.

In order to evaluate the performance of our proposed joint relational classification model, in this article, we compare our proposed model (Bi-LSTM-Attn) with the CNN model, both of which are jointly trained with RL as shown in Figure 7. It can be observed in the figure that when the recall rate is less than 0.17, our proposed model is better than CNN. When the recall rate is greater than 0.17, the CNN model performs better than our model. After our analysis, there are two reasons for this phenomenon. The first reason is that Bi-LSTM has a sequence in the time dimension, and the order of input will affect the output. CNN is more about obtaining overall information from local information aggregation and extracting hierarchical information from the input. The noise data in the test dataset are not too much at the beginning of the test. As the noise data increase, the precision decreases. Because we are using a Bi-LSTM model, in the case of not much noise data, our model has a stronger understanding of the semantics of the entire sequence than CNN, but as the amount of data and time increases, the understanding of the model will weaken. Another reason is the impact of both the size of the hidden layer and the batch size on the performance of the neural network. Due to the different sentence lengths in the dataset, the choice of hidden layer size is very important. At the same time, the size of each batch of data will also affect the model.

**Figure 8:**
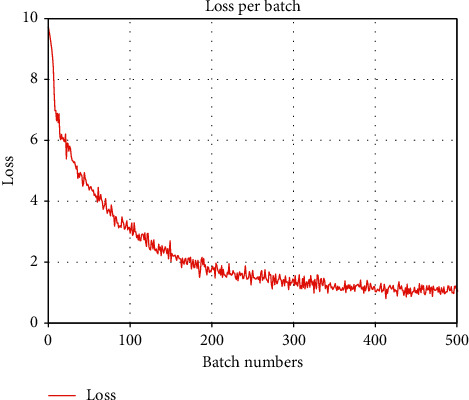
Loss per batch.

While training the model, we perform statistics on the loss value of each batch. The result is shown in Figure 8. At the beginning of training, the model has not been well trained, the loss value of each batch is relatively large, and the classification effect of the model is relatively poor. As the number of batches increases, the loss value decreases and tends to stabilize, which means that the difference between the real probability distribution and the predicted probability distribution in the training process becomes smaller, and the model's classification effect in relation classification becomes better.

In order to verify that our RL method can be beneficial to the relation classification of multisentence entity pairs, we show the P@*N* values obtained by different methods in Table 4. More specifically, we randomly select one, two, or all sentences from these test data packets to predict this relation and give P@100, P@200, and P@300 and their average values for each method. We can see that combining the attention mechanism can improve the results of the Bi-LSTM model. After combining the RL mechanism, the average value has increased by 4%, which indicates that the ability to process noisy data has improved. In particular, the Bi-LSTM-Attn-RL method achieves the best P@N results in all these settings, which are 20.6 points higher than LSTM, 12.1 points higher than Bi-LSTM, and 3.3 points higher than Bi-LSTM-Attn. For the case of using the CNN method, compared with CNN, CNN combined with the RL mechanism improves by 14%. These results show that RL can improve the sentence-level denoting effect in the Bi-LSTM model with attention mechanism.

## 6. Conclusion and Future Work

This paper proposes an RL-based distant supervision relation classification method to process noisy datasets and realizes sentence-level relation classification. In this model, the joint network model predicts the relation in the sentences selected by the RL agent and provides rewards for the RL agent. RL agent updates its strategic network based on the reward. In addition, the attention mechanism is used in the joint network model to automatically select semantic information that has a decisive influence on classification and improve the performance of the model. At the same time, the joint training of the two modules can correct the noisy labels generated by distant supervision and improve the performance of association extraction. A large number of experiments have shown that our model can filter noisy sentences well and has better sentence-level relation classification performance in noisy datasets.

In future work, we will study how to combine relation classification and NER. Entity recognition can mark the nouns in the sentence and cooperate with the joint classification network to refine the sentence structure to further understand the hidden information of the sentence.

## Figures and Tables

**Algorithm 1:**
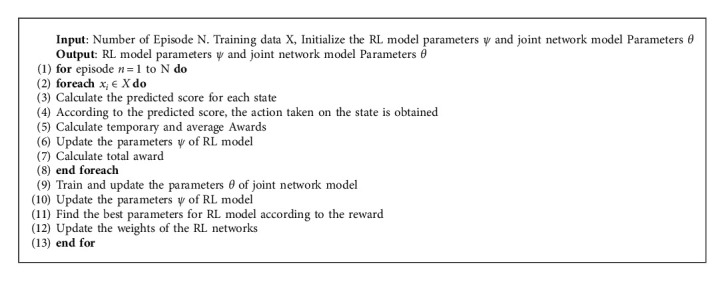
Joint training of the RL model and joint network model.

**Table 1:** Statistical dataset.

Type	Sentence	Entity pairs	Relational facts
Training set	522,611	281,270	18,252
Test set	172,448	96,678	1,950

**Table 2:** Experimental parameters.

Parameters	Values
Word dropout	0.5
Position embedding dim	5
Bi-LSTM dropout	0.75
Bi-LSTM hidden size	128
Word embedding dim	50
Batch size	128
Learning rate	0.02
Update rate	0.01
Regularization	0.0001
Sentence length	70
Sample times	3
Kernel size	60
Stride size	1
Sentence output size	230
Dropout	0.5

**Table 3:** Comparison of experimental results of each group.

Method	Precision	Recall	F1
Bi-LSTM-CRF	0.646	0.305	0.421
Bi-LSTM-Attn	0.650	0.447	0.524
Bi-LSTM	0.615	0.414	0.495
LSTM	0.608	0.392	0.488
Bi-LSTM-Attn-RL	**0.661**	**0.452**	**0.528**
Bi-LSTM-CRF-RL	0.649	0.358	0.466

**Table 4:** P@N comparison between different methods.

P@N (%)	100	200	300	Mean
LSTM	62	58	51.3	57.1
Bi-LSTM	73	65	59	65.6
Bi-LSTM-Attn	79	74	70.3	74.4
Bi-LSTM-Attn-RL	**81**	**80**	**72.3**	**77.7**
CNN	64	67	65.6	65.5
CNN-RL	78	76	70.6	74.8

## Data Availability

The data used to support this study are available in the website: https://catalog.ldc.upenn.edu/LDC2008T19.
